# Pathological Aspects of Neuronal Hyperploidization in Alzheimer’s Disease Evidenced by Computer Simulation

**DOI:** 10.3389/fgene.2020.00287

**Published:** 2020-03-27

**Authors:** Estíbaliz Barrio-Alonso, Bérénice Fontana, Manuel Valero, José M. Frade

**Affiliations:** ^1^Department of Molecular, Cellular, and Developmental Neurobiology, Cajal Institute, CSIC, Madrid, Spain; ^2^Neuroscience Institute, New York University, New York, NY, United States

**Keywords:** neuronal cell cycle reentry, SV40 large T antigen, neuron hypertrophy, neurite retraction, synaptic dysfunction, neural network modeling, synaptic firing rate, oscillatory patterns

## Abstract

When subjected to stress, terminally differentiated neurons are susceptible to reactivate the cell cycle and become hyperploid. This process is well documented in Alzheimer’s disease (AD), where it may participate in the etiology of the disease. However, despite its potential importance, the effects of neuronal hyperploidy (NH) on brain function and its relationship with AD remains obscure. An important step forward in our understanding of the pathological effect of NH has been the development of transgenic mice with neuronal expression of oncogenes as model systems of AD. The analysis of these mice has demonstrated that forced cell cycle reentry in neurons results in most hallmarks of AD, including neurofibrillary tangles, Aβ peptide deposits, gliosis, cognitive loss, and neuronal death. Nevertheless, in contrast to the pathological situation, where a relatively small proportion of neurons become hyperploid, neuronal cell cycle reentry in these mice is generalized. We have recently developed an *in vitro* system in which cell cycle is induced in a reduced proportion of differentiated neurons, mimicking the *in vivo* situation. This manipulation reveals that NH correlates with synaptic dysfunction and morphological changes in the affected neurons, and that membrane depolarization facilitates the survival of hyperploid neurons. This suggests that the integration of synaptically silent, hyperploid neurons in electrically active neural networks allows their survival while perturbing the normal functioning of the network itself, a hypothesis that we have tested *in silico*. In this perspective, we will discuss on these aspects trying to convince the reader that NH represents a relevant process in AD.

## Introduction

As the nervous system ages, it undergoes functional alterations that diminish its performance and, as these changes increase, brain homeostasis becomes compromised resulting in neurodegenerative conditions including Alzheimer’s disease (AD). A plethora of neuroanatomical and functional alterations in the nervous system accompanying the process of aging and leading to AD-associated neurodegeneration has so far been described. Among these changes, DNA level variation and aneuploidy (Cuccaro et al., 2017; Shepherd et al., 2018) as well as cell cycle reentry in neurons leading to increased DNA levels [i.e., neuronal hyperploidy (NH)] (Frade and Ovejero-Benito, 2015) are known to precede and recapitulate the classical neuropathological signs of AD (Yang et al., 2001; Arendt et al., 2010; Frade and López-Sánchez, 2017). In some cases, NH results in full DNA duplication (i.e., neuronal tetraploidy). This latter condition affects around 2–3% of neurons in AD (Mosch et al., 2007; López-Sánchez et al., 2017), a proportion that increases to around 8% when specific neuronal subtypes are evaluated (López-Sánchez et al., 2017). Once chromosomes have been fully replicated in these neurons, the latter may remain as 2N cells with 4C DNA content, as observed in G2, or as 4N cells, if they undergo premature chromosomal separation (Spremo-Potparević et al., 2008; Bajić et al., 2009). Moreover, above 30% of neurons become hyperploid in the middle stages of AD (Arendt et al., 2010). Since the fate of hyperploid neurons is delayed cell death (Yang et al., 2001; Arendt et al., 2010) these numbers likely underestimate the actual proportion of AD-affected neurons undergoing NH.

The involvement of NH in the etiology of AD has been directly proven by forcing neuronal cell cycle reentry in transgenic mice expressing oncogenes such as SV40 T large antigen (TAg) (Park et al., 2007) or c-Myc (Lee et al., 2009) under the control of the neuron-specific CAMKII promoter. This manipulation results in neuropathological hallmarks of AD, including tau protein hyperphosphorylation and neurofibrillary tangles, extracellular deposits of Aβ peptide, neuronal cell death, gliosis, and cognitive deficits. McShea et al. (2007) have also shown that c-Myc/Ras-induced cell cycle reentry in primary cortical neurons triggers tau phosphorylation that result in conformational changes similar to that seen in AD.

NH might also lead to other alterations compromising normal brain function, thus participating in several aspects of the etiology of AD (Frade and López-Sánchez, 2010). In this regard, the increase of ploidy levels is associated with nuclear and cellular hypertrophy (Orr-Weaver, 2015), and several lines of evidence suggest that these changes can be detected in AD (Frade and López-Sánchez, 2010). In this article, we will explore the morphological changes observed in cortical neurons forced to reactivate the cell cycle in response to TAg expression, a procedure recently used by our laboratory to induce hyperploidy in a small proportion of cortical neurons, thus mimicking the *in vivo* situation (Barrio-Alonso et al., 2018). By using this model, we demonstrated that neuronal hyperploidization correlates with synaptic dysfunction (Barrio-Alonso et al., 2018), a known alteration occurring at early stages of AD (Scheff et al., 2006), and that membrane depolarization with high K^+^ facilitates the survival of hyperploid neurons without reversing synaptic dysfunction in these cells (Barrio-Alonso et al., 2018). This suggests that AD-associated hyperploid neurons can be sustained *in vivo* if integrated in active neuronal circuits while remaining synaptically silent (i.e., without capacity to fire action potentials). Given that each cortical neuron can establish connections with hundreds other neuronal cells (Markram et al., 2015), it is conceivable that a relatively small fraction of silent hyperploid neurons could disrupt the normal functioning of the circuits in which they are inserted (Lusch et al., 2018). If this were the case, NH might contribute to cognitive impairment at early stages of AD due to synaptic dysfunction, while triggering neuronal cell death at later stages (Yang et al., 2001; Arendt et al., 2010; Barrio-Alonso et al., 2018). On this basis, we have also explored whether the presence of hyperploid neurons could disrupt the normal functioning of the circuits in which they are inserted. As a first approximation to the problem, this analysis has been performed *in silico*, by simulating the outcome of a neural network that contains different proportions of silent, hyperploid neurons.

## Results and Discussion

### Morphological Changes Induced by Cell Cycle Reentry in Cortical Neurons

**FIGURE 1:**
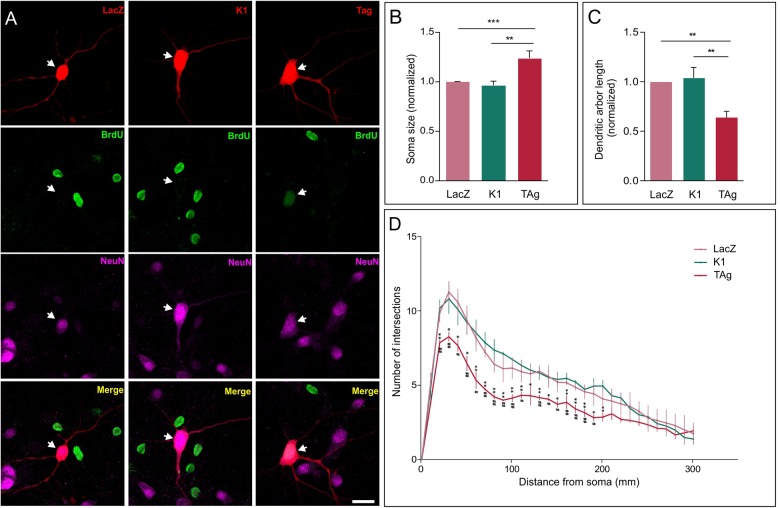
Effect of cell cycle reentry on the neuronal soma and dendritic tree. **(A)** TAg-lipofected neurons incorporating BrdU exhibit a larger soma than both control (LacZ) and K1-lipofected neurons (representative examples are shown). Arrow: neuron lipofected for 48 h with RFP and the indicated protein. Scale bar: 20 μm. **(B)** Soma size quantification in control (LacZ), K1-lipofected, and TAg-lipofected neurons, normalized to control (*n* = 3). **(C)** Normalized length of the dendritic tree in neurons lipofected for 48 h with LacZ, K1, or TAg (*n* = 3). **(D)** Average number of intersections for each distance from the soma in lipofected neurons with LacZ, Tag, or K1 (*n* = 3). Hashtags: statistical significance between LacZ and TAg; asterisks: statistical significance between K1 and TAg. ***p < 0.001, **p < 0.01, *p < 0.05, ^##^*p* < 0.01, ^#^*p* < 0.05 (One-way ANOVA followed by Tukey’s *post hoc* test in **(B,C)**; two-way ANOVA, followed by Tukey’s *post hoc* test in **(D)**.

We exploited the capacity of TAg to induce cell cycle reentry in cortical neurons (Barrio-Alonso et al., 2018) to explore the effects of hyperploidy on neuronal morphology. This analysis demonstrated that, 48 h after lipofection, the soma of cortical neurons expressing TAg was significantly larger than that of control neurons expressing either LacZ or TAg K1 (K1), an E107K TAg variant that lacks binding capacity to the pRb family members and therefore cannot induce cell cycle reactivation (Zalvide and DeCaprio, 1995; Figures 1A,B). Since no significant differences were observed between cell somas of neurons lipofected with LacZ or K1 (Figure 1B), we concluded that the effect of TAg on cell soma size is specific on its capacity to induce cell cycle reentry/hyperploidy (Barrio-Alonso et al., 2018).

We also found that, at this time point, TAg-induced cell cycle reentry specifically triggered a significant reduction in the length of the dendritic tree of cortical neurons, as compared with neurons lipofected with LacZ or K1 (Figure 1C; Supplementary Figure 1). The observed length reduction correlated with the degree of dendrite branching, evaluated through Sholl analysis, which was significantly reduced in TAg-lipofected neurons (Figure 1D). Again, this effect derives from the capacity of TAg to induce cell cycle re-entry as the K1 construct did not modified the branching profile (Figure 1D). The reduction of dendritic length and branching observed in TAg-lipofected neurons is consistent with studies carried out with mouse models of AD and postmortem material from AD patients in which a reduction in the total dendritic area was evident (Moolman et al., 2004). Interestingly, this reduction of dendritic length and branching mimics what has been observed in mitotic neurons induced to reactivate the cell cycle with a truncated form of cyclin E/Cdk2 (Walton et al., 2019).

### Simulation of Neural Networks Containing Silent Hyperploid Neurons

The morphological changes observed in neurons that reactivate the cell cycle, along with the capacity of cell cycle reentry to trigger synaptic dysfunction in neurons (Barrio-Alonso et al., 2018) suggest that NH participates in the etiology of Alzheimer by affecting neurons’ capacity to fire action potentials and therefore altering the neuronal circuits in which hyperploid neurons are inserted.

**FIGURE 2:**
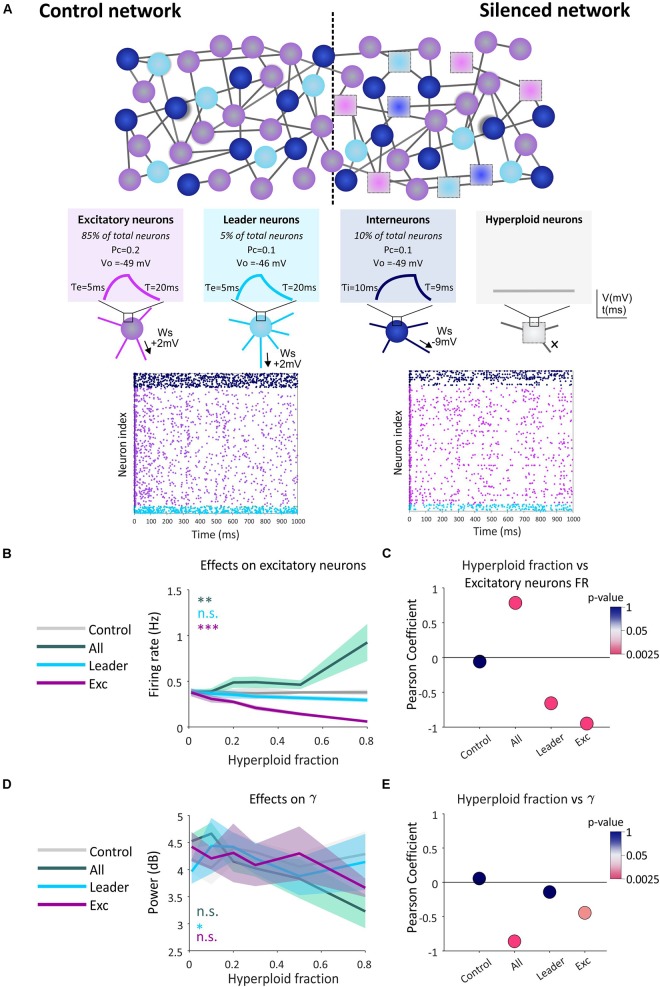
Effect of the presence of hyperploid neurons on the firing frequency of the excitatory subpopulation and in gamma type oscillations. **(A)** Hyperploidization neural network model. An example of the control network (without the presence of hyperploid neurons) is shown in comparison with a network with hyperploid neurons distributed randomly throughout the network (Silenced network). Excitatory neurons are shown in purple, interneurons in dark blue, leader neurons in light blue, and silent hyperploid neurons are shown as squares. Parameters defining the different neuronal subpopulations (*Ws*, *P*_*C*_, V_0_, τ, τ_*e*_, and τ_*i*_) are described in the methodological section. In plots, each dot indicates an action potential emitted by each neuron. The decrease in the number of dots is noticeable in the affected network with a 50% of random neuronal silencing compared to the control network. **(B)** The graph shows how the firing frequency of the excitatory neuron subpopulation is affected by the indicated fraction of silent hyperploid neurons in the whole neuron population (green), leader neurons (blue), or excitatory neurons (purple). Each line shows the average trigger frequency of each population when the corresponding type of neurons have been affected. The gray line shows the average frequency of the population when there is no silencing of neurons. 95% confidence intervals for each line are shown in shading. **(C)** Each point represents the Pearson correlation value. The color code shows the *p-*value (in logarithmic scale) of the correlation. **(D)** The graph shows how the activity of gamma rhythm is affected at the indicated fraction of silent hyperploid neurons in the whole neuron population (green), leader neurons (blue), or excitatory neurons (purple). The gray line shows the average power of this population oscillation when there is no silencing of neurons. 95% confidence intervals for each line are shown in shading. **(E)** Each point represents the Pearson correlation value. The color code shows the *p-*value (in logarithmic scale) of the correlation. **p < 0.01; ***p < 0.001 (two-way ANOVA followed by Tukey’s *post hoc* test), n.s.: non-significant, in **(B, C)**.

As a first attempt to verify this hypothesis, we employed an *in silico* approach. We simulated the impact that the presence of silent hyperploid neurons may have on the functional connectivity of a neural network through an “integrate-and-fire” model (Knight, 1972) constituted by 4,000 neurons. In this model, the membrane potential of each neuron at any simulation time point (*dt* = 1 ms) depends on two factors: (i) an exponential function, governed by a time constant, which pushes the voltage to its resting membrane potential; and (ii) the amount of excitation and synaptic inhibition received from the partner cells (Figure 2A). The local field potential (LFP) was estimated as the average of all transmembrane currents.

Different fractions of silent neurons (0.01, 0.1, 0.2, 0.3, 0.5, or 0.8) were included in (i) the whole neuronal population, (ii) the excitatory neuronal subpopulation, or (iii) the leading neuronal subpopulation, defined as those neurons with higher firing frequency and thus representing functional circuit hubs. In contrast, the control condition included no silent neurons. In a different cohort of simulations, we tested the effect of silencing the inhibitory neuron subpopulation (interneurons). As expected, this manipulation leads to an epileptic-like network with hyper-synchronous activity patterns (Supplementary Figure 2), maybe resembling the comorbidity between AD and epilepsy seen in some transgenic mouse models (Palop et al., 2007).

Our results indicate that an increase in the percentage of synaptic silencing in any of the analyzed neuronal types has defined effects on the firing rate of all simulated subpopulations present in the neural network (see Figure 2B for the subpopulation of excitatory neurons, and Supplementary Figures 3A–C for other neuronal subpopulations). In all cases, a significant inverse correlation between the fraction of silenced leading or excitatory neurons, and the firing frequency was observed (Figure 2C and Supplementary Figures 3D–F). In contrast, the disruption in the firing ability over the entire network led to an increase in the firing frequency of the excitatory subpopulation and the whole population (Figure 2C and Supplementary Figure 3F), likely due to silencing of a portion of inhibitory neurons. Therefore, we concluded that the presence of hyperploid neurons with synaptic deficits (silent neurons) affects the firing frequency of the neural network in which they are integrated. This effect is cell-type dependent and correlated to the fraction of affected neurons, causing an increase or decrease of firing rate depending on the tested cell type.

We also explored the effect of silencing on the oscillatory patterns generated spontaneously by the network. Brain oscillations, similar to those observed in our model, are an emergent property of the system associated with the coordination of the circuit and the transmission of information between its elements (Buzsáki and Draguhn, 2004; Buzsáki et al., 2013). In this regard, our model presents peaks of synaptic activity in the spectral bands gamma, beta, theta and delta (Buzsáki and Draguhn, 2004; Supplementary Figure 4). We observed a significant effect of synaptic silencing on gamma activity, this being dependent on both the proportion of silent neurons and the specific population in which they are found (Figure 2D). These effects are mainly appreciated as a negative correlation between silencing in total and excitatory neurons and gamma power (Figure 2E), reminiscent of the gamma oscillations impairment observed in AD (Mably and Colgin, 2018). In the rest of the spectral bands we found minor or insignificant changes except for the whole neuronal population and the excitatory subpopulation when the beta and theta rhythms, respectively, were analyzed (Supplementary Figure 5).

## Conclusion

Cumulative evidence indicates that NH may participate in the classical neuropathology observed in AD. In addition, our results suggest that NH can also lead to alterations in neuronal circuit functioning due to the morphological and synaptic changes observed in hyperploid neurons. We believe these alterations, together with any other perturbation underlying the synaptic deficits found in AD (Scheff et al., 2006), could account for the etiology of AD as well. Our simulation study indicates that NH may trigger alterations in the firing frequency of the neural network, an effect that increases as the proportion of hyperploid neurons raises. Therefore, the presence of a high proportion of hyperploid neurons in specific local circuits could lead to major effects in AD. This conclusion should be experimentally tested in the future. In areas such as the entorhinal cortex, where above 30% of neurons become hyperploid in AD patients (Arendt et al., 2010), this condition could have an important impact not only on the firing frequency but also on the oscillations observed in the neural networks (Kitchigina, 2018), which according to our *in silico* model requires a high proportion of silent neurons to be relevant.

## Materials and Methods

### Neuronal Soma and Dendritic Tree Analysis

Primary cortical cultures, co-lipofection with red fluorescent protein (RFP) and plasmids expressing LacZ, TAg, or K1, and immunocytochemistry were performed as described by Barrio-Alonso et al. (2018). RFP-positive neurons were randomly chosen. Image analysis was performed with ImageJ (National Institutes of Health). Sholl analysis (Binley et al., 2014) was carried out with the Sholl analysis module (Fiji) using digital tracings generated with the NeuronJ plugin (Fiji) from confocal projection images of neurons co-lipofected with RFP (*n* = 30 per condition). Analysis parameters were: starting radius = 1 μm, ending radius = NaN, and radius step size = 10 μm. Linear Sholl plots were generated, representing the average number of intersections with radii in each condition. Total length of neurites was also evaluated. At least 25 lipofected neurons/culture from 3 independent cultures were analyzed for each experimental condition.

### Neural Network Simulation

“Integrate-and-fire” simulation (Knight, 1972) of neural networks containing hyperploid neurons was implemented using the Python-based Brian 2 simulator (Goodman and Brette, 2008; Stimberg et al., 2017). In the simulation model (Figure 2A), each neuron has a membrane potential (V) governed by the following differential equation:

$$\tau \,\frac{\text{d}\,V}{\text{dt}}=-\left(V-V_{0}\right)\,$$

where τ is the membrane time constant, which parameterizes the time it takes for the neuron to reach its resting membrane potential (*V*_0_). In turn, *V* can be disturbed by depolarizing and hyperpolarizing synaptic currents. The excitatory and inhibitory conductances (ge and gi) of such synaptic currents follow the following differential equations:

$$\frac{\text{dge}}{\text{dt}}=-\frac{\text{ge}}{\tau \,e}\,$$

$$\frac{\text{dgi}}{\text{dt}}=-\frac{\text{gi}}{\tau \,i}\,$$

The value of ge and gi depends, respectively, on the number of active excitatory and inhibitory synapses according to their synaptic weight (*Ws*). The *Ws* of each excitatory synapse is +2 mV while each inhibitory synapse has a *Ws* of −9 mV. An action potential (AP) from synapse *s* at time *t* induces the following change in V in neuron *j*: *V*→ *V* + *Ws*. Thus, if *s* is an excitatory synapse, the neuron *j* is depolarized by +2 mV at a rate defined by τe. An inhibitory synapse induces a change of −9 mV governed by τi.

Each neuron initializes V in a randomly chosen value between −50 and −60 mV and begins to receive excitatory and inhibitory synapses with their corresponding weights. If V reaches −50 mV, the trigger AP threshold (Vt), the neuron fires, which induces a synaptic current in those neurons to which it is connected. At this point, there is a refractory period of 5 ms in which the neuron cannot fire again.

The network is composed of 4,000 neurons of three types, with different proportion, electrical properties, and probabilities of connection to other neurons (*Pc*): (i) *excitatory neurons* (85% of all neurons), τe = 20 ms, V_0_ = −49 mV, and *Pc* = 0.1; (ii) *leading neurons* (5% of all neurons), a subtype of excitatory neurons whose membrane potential is 3 mV more depolarized, and therefore have a higher trigger frequency that simulate neurons constituting relevant hubs of the circuit; and (iii) *interneurons* (10% of all neurons), τi = 9 ms (i.e., high trigger frequency), V_0_ = −49 mV, and *Pc* = 0.2. Five repetitions of each condition were simulated.

Parameters established in the model reasonably mimic the physiological situation (Markram et al., 2015) according to the firing frequency: most excitatory neurons fires at a frequency lower than 1 Hz, leading neurons fire at a frequency of 1–5 Hz, and the inhibitory neurons (or interneurons) show a firing frequency of around 2 Hz.

For the simulation of hyperploid neurons (i.e., silent neurons unable to fire APs), a “damage” parameter (*dam*) was incorporated in the differential equation of the model. *dam* has a value equal to −30 mV, which hyperpolarizes the membrane potential, setting its *V* away from *Vt*. The percentages of hyperploid neurons in this study were 1, 10, 20, 30, 50, and 80%. This provides a complete picture of the effects of this variable on the outcome of the neural network.

As an internal control, we found that the simulated neural network has a strong synaptic dependence on its activity patterns. If all synaptic connections are removed, the network presents a synchronous firing rate, reflecting the exponential component that repolarizes V (Supplementary Figure 6).

Oscillatory patterns and their power were estimated by the sum of all membrane potentials from the network (Supplementary Figure 4).

### Statistical Analysis

Statistical analyses were performed with ANOVA, followed by the Tukey’s *post hoc* test. Pearson correlation test was also applied in simulation experiments.

## Data Availability Statement

The complete code for the simulation is in the Supplementary Material. Data were analyzed with MATLAB (MathWorks). The data and the MATLAB scripts used to generate the analyses and representations can be downloaded from http://dx.doi.org/10.20350/digitalCSIC/10541.

## Ethics Statement

This study was carried out in accordance with the principles of the Basel Declaration and the EU guidelines for the care and use of laboratory animals. All of the procedures for handling and sacrificing animals were approved by the CSIC Ethics Committee.

## Author Contributions

JF conceived the study and wrote the manuscript. EB-A and MV designed the neural network, performed the simulations and analyzed the data. EB-A also contributed to the manuscript writing. BF carried out the neuronal soma and dendritic tree analysis, EB-A and BF prepared figures. All authors approved the final manuscript.

## Conflict of Interest

JF is shareholder (ten percent equity ownership) of Tetraneuron, S.L., a biotech company exploiting his patent on the blockade of neuronal tetraploidy as a therapeutic approach against AD and funding his work on therapies against AD based on neuronal tetraploidy blockade. The remaining authors declare that the research was conducted in the absence of any commercial or financial relationships that could be construed as a potential conflict of interest.
