# Supplementary material for: Pathological Aspects of Neuronal Hyperploidization in Alzheimer’s Disease Evidenced by Computer Simulation
Source: Front Genet. 2020 Mar 27;11:287. doi: 10.3389/fgene.2020.00287 (PMC7121139; doi:10.3389/fgene.2020.00287)
Supplement: Supplementary file 1 [file Presentation_1.pdf]

Supplementary Material

Code used with the Brain 2 simulator

```
########################################
######### HYPERPLOIDY_MODEL V1 #########
########################################

# loading packages
from brian2 import *
%matplotlib inline
from brian2tools import *
import numpy, random, warnings, time, os
import pandas as pd
warnings.filterwarnings('ignore')
start_scope()
# Damage network
Prob_tet = 0.8 # percentage of non-spiking cells (over size network,
independent of type)
Cell_tet = 'all' # all, lead, exc, inh
no_syn = False # No synapsis
# Parameters to play with
N = 4000 # Network size
dur = 1 # simulation duration, in seconds
taum_e = 20*ms # membrane time constant
taum_i = 9*ms # membrane time constant (not 10, because eqs does 0)
Perc_inh = .1 # percentage of inhibitory neurons
Perc_lead = .05 # percentage of leader neurons
prob_Ce = 0.1 # excitatory connection probability
prob_Ci = 0.2 # inhihibitory connection probability
prob_Cl = 0.1 # leader connection probability
we = 2*mV # excitatory synaptic weight (voltage)
wi = -9*mV # inhibitory synaptic weight
El_h = -49*mV # membrane potential, for all neurons
El_l = -46*mV # membrane potential, for leaders
```

```
# Network parameters
taue = 5*ms # excitation time constant
taui = 10*ms # inhibition time constant
Vt = -50*mV # spike threshold
Vr = -55*mV # reset threshold
# Internal variables
Exc_sz = int(N - (N * Perc_inh)) # Size of excitatory population
Leader_sz = int(N * Perc_lead) # Size of leadership population
Dam_sz = int(N*Prob_tet) # Size of damage population (non-spiking
cells)
if no_syn:
prob_Ce = 0
prob_Ci = 0
prob_Cl = 0
eqs = '''
dv/dt = (ge+gi-(v-El-dam))/tau : volt (unless refractory)
dge/dt = -ge/taue : volt
dgi/dt = -gi/taui : volt
tau : second
El : volt
dam : volt
'''
P = NeuronGroup(N, eqs, threshold='v>Vt', reset='v = Vr',
refractory=5*ms,method='exact')
P.v = 'Vr + rand() * (Vt - Vr)' # initial v, random bet -60 to -50
P.ge = 0*mV # initial G on 0
P.gi = 0*mV
P.tau[:Exc_sz] = taum_e # tau for excitatory neurons
P.tau[Exc_sz:] = taum_i # tau for inhibitory neurons
P.El = El_h
P.El[:Leader_sz] = El_l # membrane potential for leaders
P.dam = 0 # damage parameter set as 0 for all neurons
# damage
if Prob_hip == 0:
print('Control hipModel')
damp_pop = [] # damage population
Cell_hip = 'Control'
code = 0
```

```
elif Cell_hip is 'all':
damp_pop = random.sample(list(range(N)),Dam_sz)
print('{0}%hyperploids cells, entire
population'.format(Prob_hip*100))
code = 1
elif Cell_hip is 'lead':
if Dam_sz > Leader_sz: Dam_sz = Leader_sz # taking care of sizes
damp_pop = random.sample(list(range(Leader_sz)),Dam_sz)
print('{0}%hyperploids leader cells,
'.format(Dam_sz/Leader_sz*100))
code = 2
elif Cell_hip is 'exc':
if Dam_sz > Exc_sz: Dam_sz = Exc_sz # taking care of sizes
damp_pop = random.sample(list(range(Exc_sz)),Dam_sz)
print('{0}%hyperploids exc cells'.format(Dam_sz/Exc_sz*100))
code = 3
elif Cell_hip is 'inh':
if Dam_sz > N - Exc_sz: Dam_sz = N - Exc_sz # taking care of sizes
damp_pop = random.sample(list(range(Exc_sz,N,1)),Dam_sz)
print('{0}%hyperploids inh cells'.format(Dam_sz/(N - Exc_sz)*100))
code = 4
if len(damp_pop)>0: P.dam[damp_pop] = -30*mV # implement damage
Ce = Synapses(P, P, on_pre='ge += we') # Definition of an excitatory
synapsis
Ci = Synapses(P, P, on_pre='gi += wi') # Definition of an inhibitory
synapsis
Cl = Synapses(P, P, on_pre='ge += we') # Definition of an excitatory
leader neuron synapsis
Ce.connect(condition='i<Exc_sz', p=prob_Ce) # Excitatory synapsis from
definition, to all cells
Ci.connect(condition='i>=Exc_sz', p=prob_Ci) # Inhibotory synapsis
from definition, to all cells
Cl.connect(condition='i<Leader_sz',p=prob_Cl) # Leader synapsis from
definition, to all cells
spk_mon = SpikeMonitor(P)
state_mon = StateMonitor(P, 'v', record=True) # record three cells
print('HipRunning...')
run(dur * second)
print('HipDone!')
```

```
# Inspect simulation
f1=figure(figsize=(10, 4)) # Plotting raster
plot(spk_mon.t[numpy.where(spk_mon.i<Exc_sz)[0]]/ms,
spk_mon.i[numpy.where(spk_mon.i<Exc_sz)[0]], ',b') # blue are exc
plot(spk_mon.t[numpy.where(spk_mon.i<Leader_sz)[0]]/ms,
spk_mon.i[numpy.where(spk_mon.i<Leader_sz)[0]], ',g') # green are lead
plot(spk_mon.t[numpy.where(spk_mon.i>=Exc_sz)[0]]/ms,
spk_mon.i[numpy.where(spk_mon.i>=Exc_sz)[0]], ',r') # red are inh
plot([-10]*len(damp_pop),damp_pop, ',k')
xlabel('Time [ms]'), ylabel('Neuron index'), xlim(-50,dur*1000 + 50),
ylim(0-200, 4000+200)
- 170 -
print('Firing rate of Exc Lead:
{0}'.format(len(spk_mon.t[numpy.where(spk_mon.i<Leader_sz)[0]])/Leader
_sz/dur))
print('Firing rate of Exc:
{0}'.format(len(spk_mon.t[numpy.where(spk_mon.i<Exc_sz)[0]])/Exc_sz/du
r))
print('Firing rate of Inh:
{0}'.format(len(spk_mon.t[numpy.where(spk_mon.i>=Exc_sz)[0]])/(NExc_
sz)/dur))
f2=figure(figsize=(10, 4)) # Plotting some traces
for ii in random.sample(list(range(Exc_sz)),3): # plotting some exc
plot(state_mon[ii].t/ms, state_mon.v[ii].T, 'b')
for ii in random.sample(list(range(Exc_sz,N,1)),3): # plotting some
exc
plot(state_mon[ii].t/ms, state_mon.v[ii].T, 'r')
xlabel('Time [ms]'), xlim(-50,dur*1000 + 50), ylabel('mV')
print('HipSaving...')
sim_name = 'tetSim ' + Cell_tet + '_' + time.asctime() # Simulation
name
sim_name = sim_name.replace(' ','_')
sim_name = sim_name.replace(':','_')
os.mkdir(sim_name) # creating simulation folder
spk = pd.DataFrame([list(spk_mon.i), list(spk_mon.t/ms)]) # saving
spikes
spk.to_csv(sim_name + '/spk.cvs', sep=',', encoding='utf-8',
index=False, header=False)
```

```
mv = pd.DataFrame(column_stack((transpose(state_mon.v/mV),
state_mon.t))) # saving membrane potential
mv.to_csv(sim_name + '/mv.cvs', sep=',', encoding='utf-8',
index=False, header=False)
feat = {'N': [N], 'dur':[dur], 'taum_e':[taum_e/ms],
'taum_i':[taum_i/ms], 'Perc_inh':[Perc_inh],
'Perc_lead':[Perc_lead],'prob_Ce':[prob_Ce],
'prob_Ci':[prob_Ci], 'prob_Cl':[prob_Cl], 'we':[we/mvolt],
'wi':[wi/mvolt], 'El_h':[El_h/mV],
'El_l':[El_l/mV],'Perc_dam':[Prob_hip], 'type_dam':[code],
'taue':[taue/ms],
'taui':[taui/ms], 'Vt':[Vt/mV], 'Vr':[Vr/mV]} # saving
simulation parameters
feat = pd.DataFrame.from_dict(feat)
feat.to_csv(sim_name + '/feat.cvs', sep=',', encoding='utf-8',
index=False)
cellType_log = array([0] * N) # creating cell log, 0 is excitatory
cell
cellType_log[:Leader_sz] = 1 # 1 is leader
cellType_log[Exc_sz:] = 2 # 2 is inh
hip_log = array([0] * N) # creating hip log, 0 is healthy cell
hip_log[damp_pop] = 1 # 1 is hip
log = pd.DataFrame(column_stack((cellType_log,
tet_log)),columns=['cellType','Tet'])
log.to_csv(sim_name + '/log.cvs', sep=',', encoding='utf-8',
index=False)
f1.savefig(sim_name + '/raster.png') # Saving figures
f2.savefig(sim_name + '/SomeTraces.png')

print('TetSaved. Thanks for using HipModel v1.')
```

Supplementary Figures

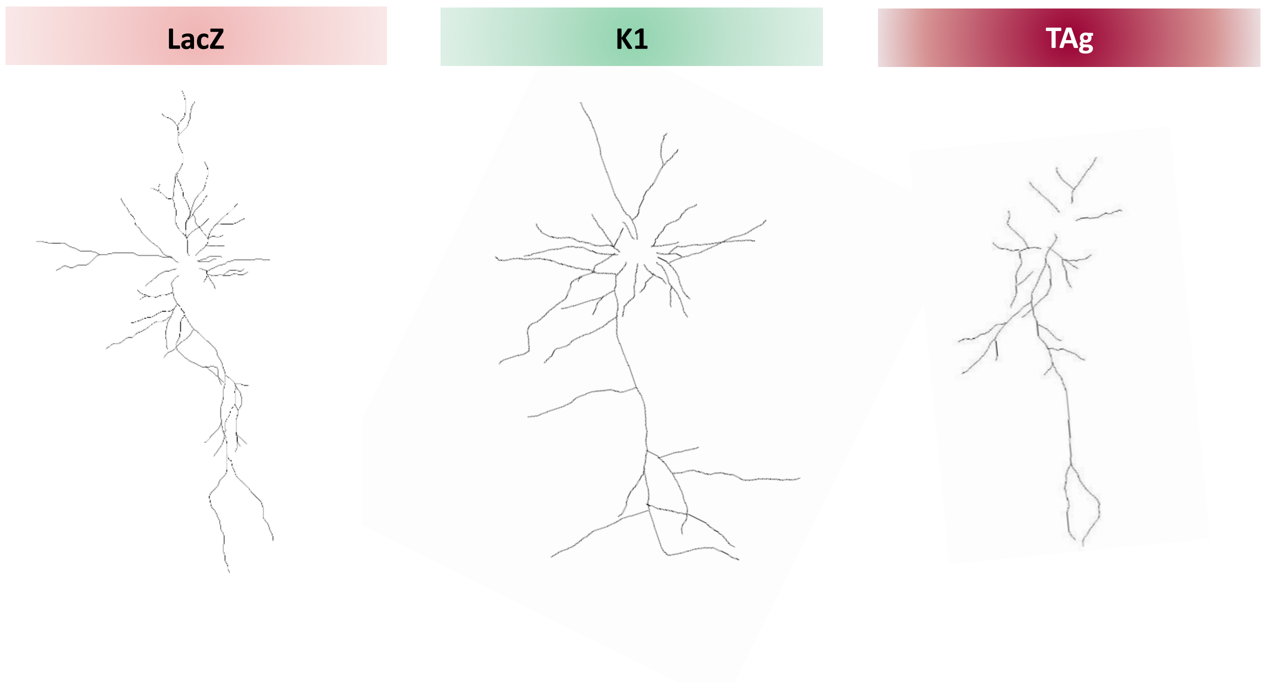

Supplementary Figure 1. Representative diagram of the dendritic tree of neurons lipofected with LacZ, K1, or TAg (see Fig. 2C).

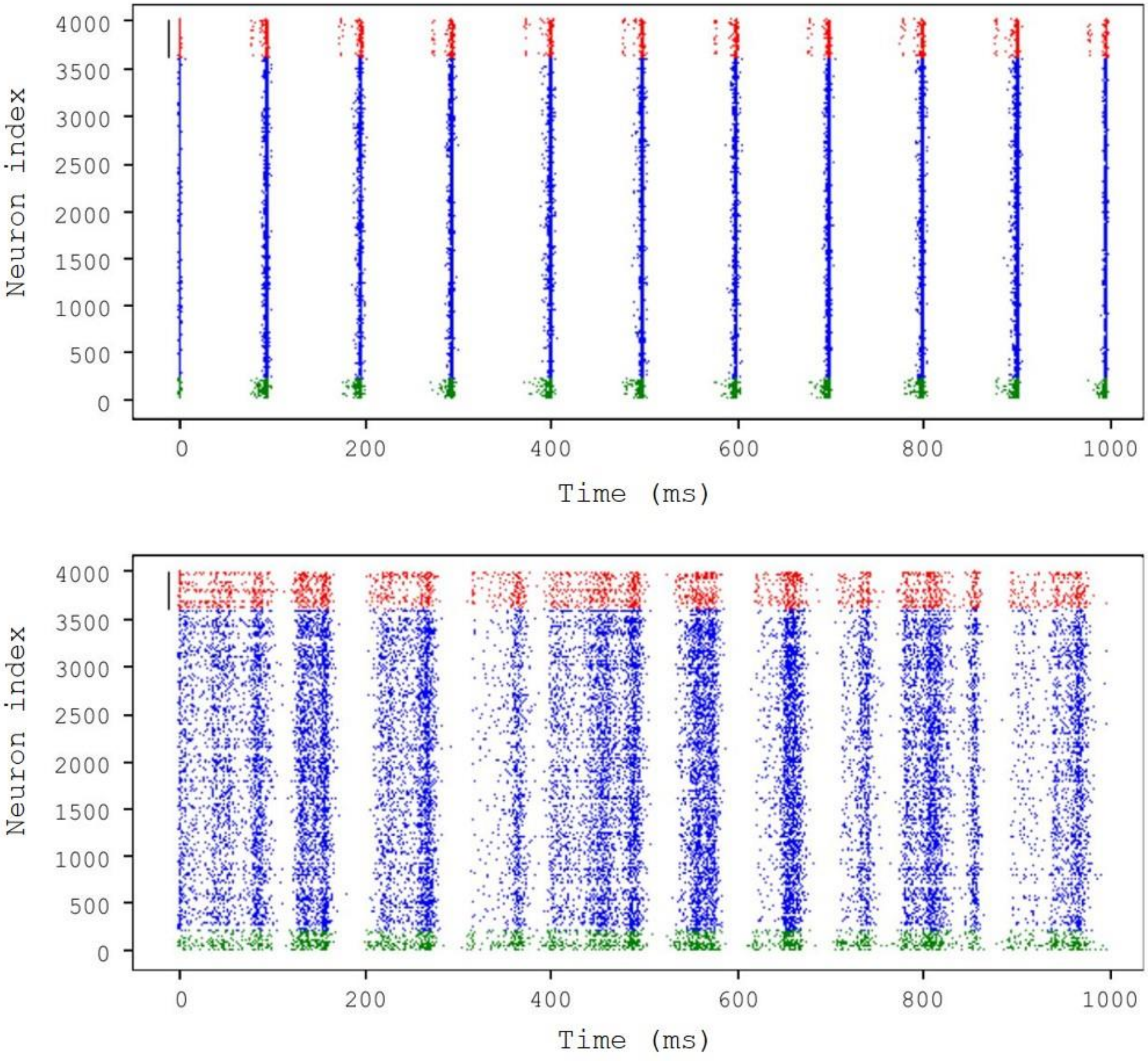

Supplementary Figure 2. Silencing of the interneuron subpopulation results in an epileptic-like network with synchronous activity patterns. Representative examples of simulations with 50% (upper panel) and 80% (lower panel) of hyperploid neurons in the interneuron subpopulation. Synchronous firing patterns of epileptic type are observed. Each point of the graph represents AP triggers of interneurons (red), excitatory neurons (blue) and leading neurons (green).

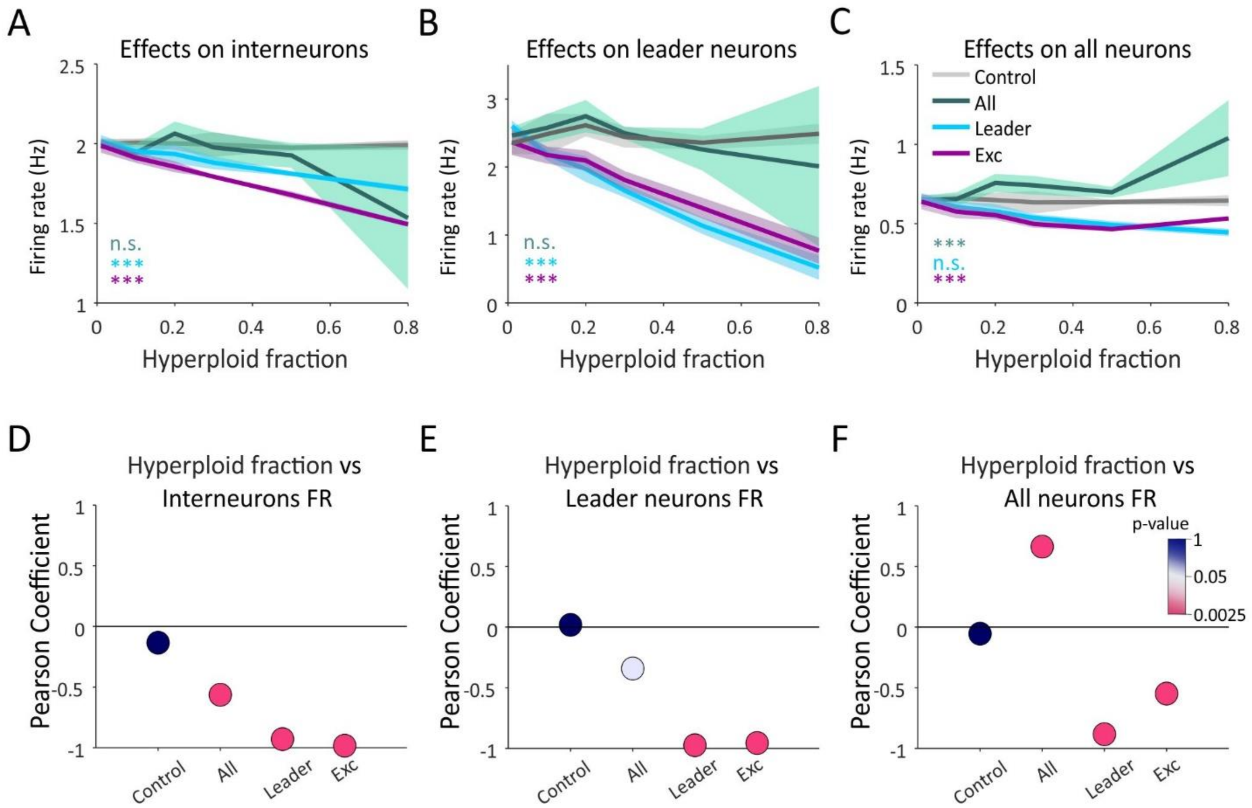

Supplementary Figure 3. Effect of the presence of hyperploid neurons on the firing frequency of different neuronal subpopulation. The graph shows how the firing frequency of the subpopulation of interneurons **(A)**, leader **(B)** and the whole population **(C)** is affected by the indicated fraction of silent hyperploid neurons in the whole neuron population (green), leader neurons (blue), or excitatory neurons (purple). Each line shows the average trigger frequency of each population when the corresponding type of neurons have been affected. The gray line shows the average frequency of the population when there is no silencing of neurons. 95% confidence intervals for each line are shown in shading. ***p<0.001 (ANOVA followed by Tukey's post-hoc test), n.s.: non-significant. **(D-F)** Each point represents the Pearson correlation value. The color code shows the p value (in logarithmic scale) of the correlation.

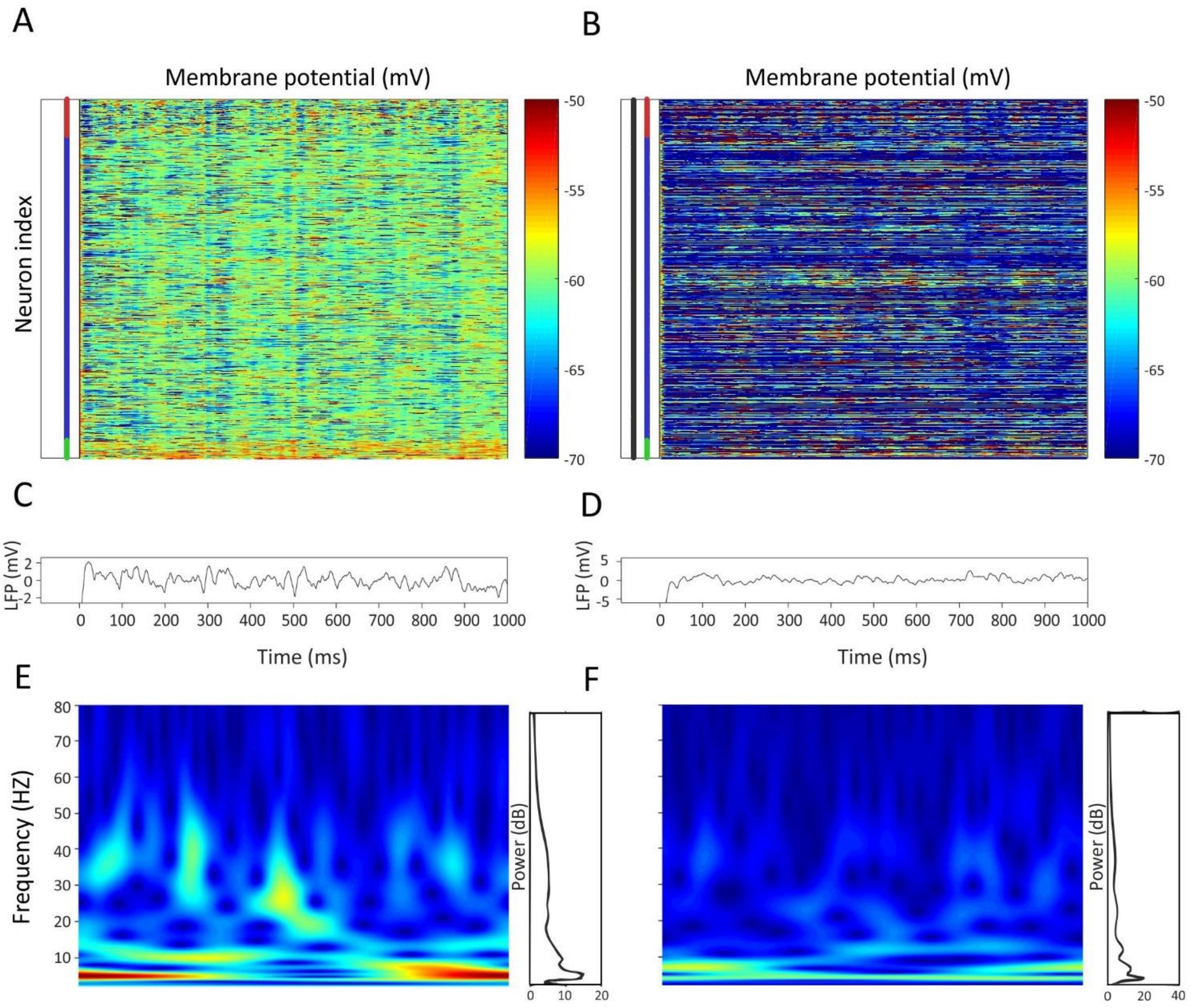

Supplementary Figure 4. Membrane potentials of all the neurons throughout the simulation (1 second), profiles of the local field potentials, and spectra of the oscillations generated from of these networks. (A, B) A representative example of membrane potentials of the control network (without the presence of hyperploid neurons) is shown (left) in comparison with a network with hyperploid neurons distributed randomly throughout the network (50% of silent neurons) (right). Blue tones refer to hyperpolarized membrane potentials and red tones mean depolarization values. **(C, D)** Profiles of local field potentials (mV). **(F, G)** Spectra of the oscillations generated from each network as well as the power profile. Notice the gamma bands (frequency between 30-80 Hz), beta (12-30 Hz), theta (4-8 Hz) and delta (0.1-4 Hz).

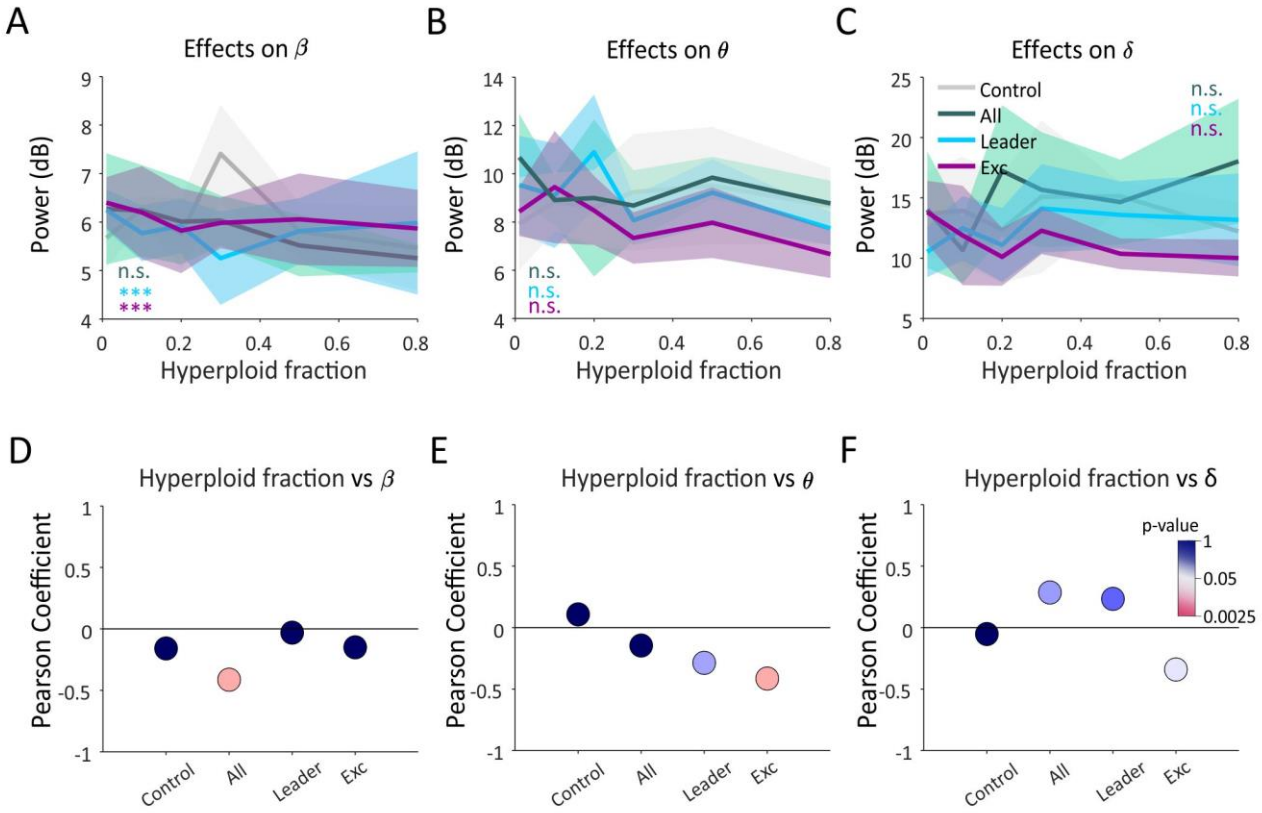

Supplementary Figure 5. Effect of the presence of hyperploid neurons in different types of oscillations. The graphs show how the activity of beta **(A)**, theta **(B)** or delta **(C)** rhythms is affected at the indicated fraction of silent hyperploid neurons in the whole neuron population (green), leader neurons (blue), or excitatory neurons (purple). The gray line shows the average power of these population oscillations when there is no silencing of neurons. ***p<0.001 (ANOVA followed by Tukey's post-hoc test), n.s.: non-significant. **(D-F)** Each point represents the Pearson correlation value. The color code shows the p value (in logarithmic scale) of the correlation.

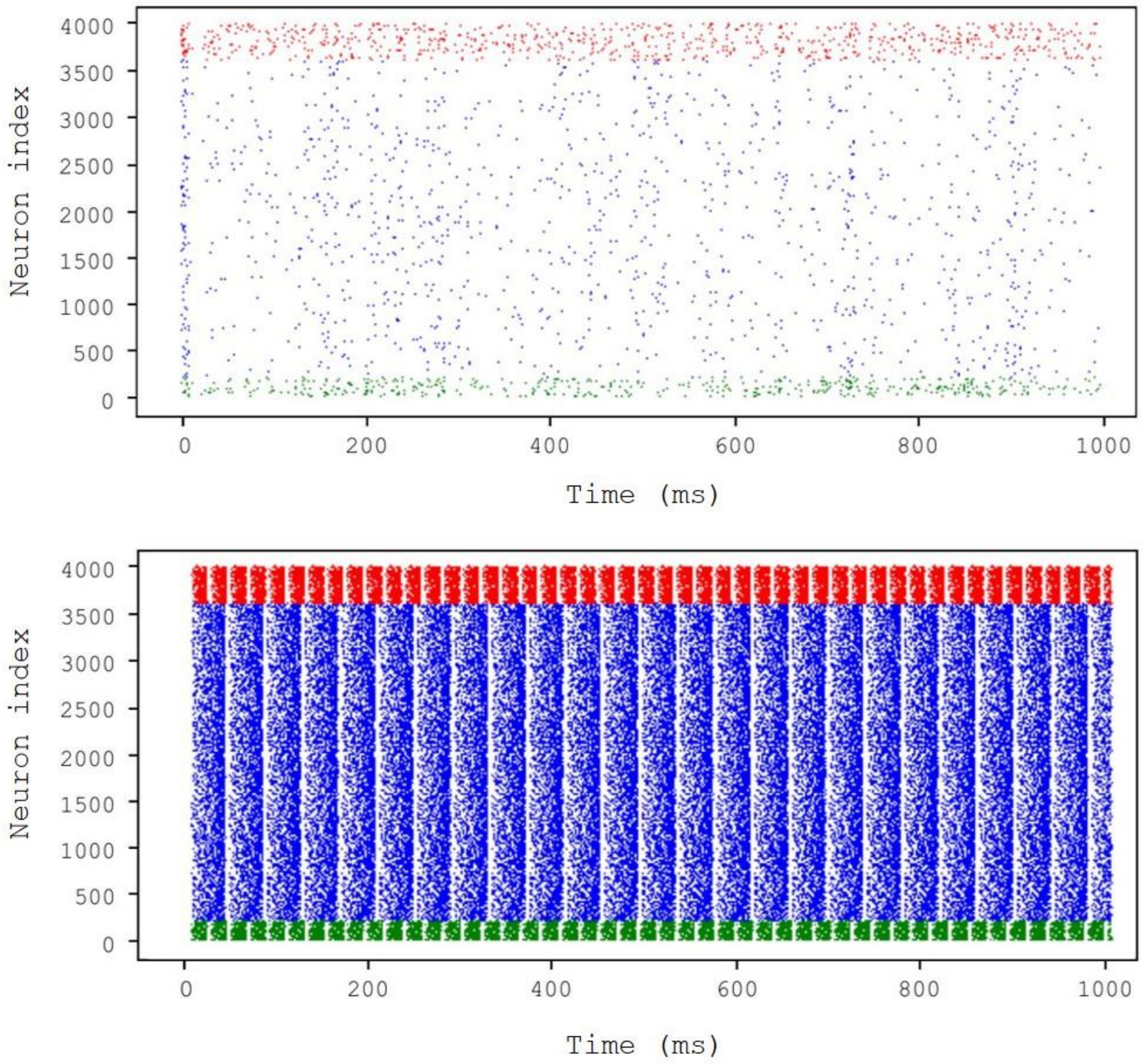

Supplementary Figure 6. The outcome of the simulated neural network is intrinsically dependent on the configured synapses. Each individual dot of the graph represents the AP trigger of a neuron (listed from 1 to 4,000 on the ordinate axis) throughout the simulation time (1,000 ms). A control simulation (upper panel) is compared with a simulation in which all the synapses have been eliminated (lower panel). Red: interneurons, blue: excitatory neurons, green: leading neurons.
